# Entrustable professional activities for Junior Brazilian Medical Students in community medicine

**DOI:** 10.1186/s12909-022-03762-4

**Published:** 2022-10-25

**Authors:** Ieda Francischetti, Ylva Holzhausen, Harm Peters

**Affiliations:** 1 Dieter Scheffner Center for Medical Education and Educational Research Charité - Universitätsmedizin Berlin, Dean?s Office of Study Affairs, Campus Charité Mitte, Charitéplatz 1, 10117 Berlin, Germany; 2 Marília Medical School (Faculdade de Medicina de Marília - FAMEMA), Marília, São Paulo, Brazil

**Keywords:** Entrustable professional activities, Undergraduate medical education, Community medicine

## Abstract

**Background:**

Entrustable professional activities (EPAs) have been defined to promote the workplace participation of undergraduate medical students, generally in the context of high-income countries with a focus on the secondary and tertiary health care sectors. These EPAs have limited applicability to training and health care contexts in low- to middle-income countries that have a focus on primary health care, for instance, the context of community medicine. The purpose of this article is to report the process and results of defining EPAs for undergraduate medical training in a community health care setting.

**Methods:**

A modified Delphi study was performed to develop EPAs for the training of medical students in community medicine during their first and second years of education at the Marília Medical School (FAMEMA), Brazil. The supervision level was operationalized in terms of a student’s ability to perform the EPA autonomously in an effective and safe manner with supervision readily available on request. Panellists (9 physicians and 6 nurses) rated the completeness of the proposed list of EPAs and EPA categories on four-point Likert scales. The threshold for consensus among panellists was a mean content validity index of at least 80%.

**Results:**

Consensus was reached after two Delphi rounds, resulting in 11 EPAs for undergraduate medical education and training in community medicine. These EPAs were organized into three overarching EPA domains: integrality of care for individual health needs in all phases of the life cycle (5 EPAs), integrality of care for family health needs (3 EPAs), and integrality of care for community health needs (3 EPAs). For each EPA, descriptions of the following categories were created: title; specifications and limitations; conditions and implications of the entrustment decision; knowledge, skills, and attitudes; links to competencies; and assessment sources.

**Conclusion:**

The resulting 11 EPAs for training medical students in community medicine expand the application of the EPA framework to both early undergraduate medical education and the context of primary health care. This report can support and guide other medical schools in their attempts to train students in primary health care contexts and to incorporate EPAs into their curricula.

**Supplementary Information:**

The online version contains supplementary material available at 10.1186/s12909-022-03762-4.

## Background

The concept of entrustable professional activities (EPAs) is utilized increasingly to operationalize competency-based education. EPAs provide a tangible means of facilitating the participation of medical trainees, including both post- and undergraduate trainees, in the patient care in various health care settings [1–5]. The transferability of previously reported EPAs used for undergraduate medical education appears to be limited, as EPAs have been developed to facilitate the workplace participation of medical students in the context of high-income countries with a focus on the secondary and tertiary health care sectors [6–14]. However, both in the literature and in practice, there is a lack of EPAs for training students in the context of low- to middle-income countries with a focus on the primary health care sector. A branch of medicine called community medicine represents one of the main primary health care-focused approaches to the comprehensive care of patients, families and society in many such countries [15–18]. EPAs for community medicine are likely to facilitate the more tangible operationalization of the health practices involved in community medicine, thereby promoting health care and improving the teaching-learning process for both students and their supervisors [4, 19, 20]. The purpose of this article is to report the process and results of defining EPAs for undergraduate medical training in a community health care setting.

The concept of EPAs was introduced in 2005 by ten Cate as an approach to improving the translation of competency-based medical education into workplace practice [21]. The concept is focused on professional activities as represented by authentic units of clinical work, and these activities are gradually entrusted to medical trainees by supervisors depending on the trainees’ level of competence. EPAs function well as educational outcomes at all stages of medical training. With regard to learning, EPAs synthesize the necessary knowledge, skills and attitudes that must be learned to carry out certain professional activities. With regard to assessment, EPAs simultaneously allow for the evaluation of acquired competencies by observable and measurable means and methods [21–23]. The concept of EPAs was first introduced for postgraduate medical training, and an increasing number of programmes worldwide have adopted this approach [24]. More recently, the concept of EPAs has been applied to undergraduate medical education with a focus on defining end-of-undergraduate training outcomes; EPAs used for this purpose are generally referred to as EPAs for entry into residency. To date, the use of EPAs for entry into residency has been reported at the national level for the USA, Canada and Switzerland and at the institutional level for Utrecht, The Netherlands, and Berlin, Germany [6, 7, 21–27]. Another set of EPAs has been defined for the participation of students in early clerkships [28, 29].

One common element among EPAs reported to be in use for undergraduate medical training is that they focus on patient care at the secondary and tertiary health care levels, that is, on care that is often specialized care and that is provided by specialist and subspecialist professionals, respectively [30, 31].However, primary health care has received less attention. The focus on secondary and tertiary health care reflects the main context of workplace learning and the participation of trainees in the high-income countries in which EPAs have been developed. Accordingly, training takes place primarily in the medical centres of universities, affiliated hospitals or specialized ambulatory clinics featuring intermediate to highly technical and specialized levels of care. Clinical work is focused on care for individual patients and entails the provision of specialized services to diagnose and treat a spectrum of disorders with a medium-to-high level of complexity, which often feature unusually severe, complex or uncommon health problems [23–27].

In contrast, the health care systems of low- to middle-income countries focus on the measures and structures involved in the primary health care sector. In a number of countries, community medicine represents the main modality for implementing primary health care [32–36]. In particular, community medicine is concerned with the promotion of health and the prevention of disease among individuals (including healthy individuals, individuals with diseases, and individuals with disabilities), families and communities. Community medicine aims to provide comprehensive health services, which include preventive, promotive, curative and rehabilitative services throughout the human life cycle, i.e., from childhood to old age. Community medicine encompasses the comprehensive planning and provision of public health services and, when necessary, the management of access to higher-level medical care [36–40]. Furthermore, the workplace participation of medical students at an early stage is common in community medicine [41–43]. Given these differences in the context and scope of health care, it may be unsurprising that the previously reported EPAs used in undergraduate medical education appear not to be sufficiently transferable or applicable to the context of training in community medicine [2, 3, 6–14, 25, 26]. While community medicine has adopted a competency-based approach to medical education, a shift towards operationalization via the application of EPAs has not yet been carried out [40, 41]. The elaboration of community medicine EPAs for undergraduate medical training would represent a first and important step in this direction. Such EPAs can define tasks for the participation of medical students in a community medicine setting, the scope and breadth of these tasks and the anticipated levels of supervision required. Therefore, community medicine EPAs are better able to operationalize expectations for students and their supervisors, and they can be utilized in curriculum development to refine and improve the alignment of the teaching, learning and assessment of medical students with respect to practice in community medicine.

The aim of this paper is to report the process and results of defining a set of community medicine EPAs for undergraduate medical training. The tasks and content of these EPAs were defined through a systematic, Delphi study-based process [44–46]. The EPAs were created based on the competency-based curriculum of a Brazilian state-operated medical school, in which students in their first and second years of education actively participate in the provision of community medicine services in affiliated family health care units [41–43, 47].

## Methods

### Setting

The study was conducted at Marília Medical School (Faculdade de Medicina de Marília - FAMEMA) and 20 affiliated Family Health Strategy (FHS) units located in Marília, a city in the state of São Paulo, Brazil, from February 2019 to October 2019. The study protocol was approved by the Brazilian Ethics and Research Commission, 3.571.405; National Health Commission 466/2012; Operating Number 001/2013. This study focuses on existing workplace placements of FAMEMA students in the field of community medicine.

FAMEMA is a public medical and nursing school. The undergraduate medical programme conducts annual enrolment, takes six years to complete and requires a total of 11.079 h of teaching and practical training [47]. The undergraduate education is followed by postgraduate training in various medical disciplines. The medical curriculum is competence-based and includes defined learning objectives for knowledge, skills, attitudes and competencies throughout all study phases; however, thus far, no EPAs have been established for the programme. In Brazil, FAMEMA is known for its pioneering adoption of the problem-based learning (PBL) approach, which it has employed for more than 24 years [48]. The FAMEMA medical curriculum includes four major longitudinal tracks [47, 49]. 1) In the Pedagogical Support Unit track, which takes place from years 1 to 6 of education, the students take classes in basic and clinical science, communication and information technology. 2) The PBL track, which is called the Systematized Education Unit, involves a total of 69 written cases and takes place from years 1 to 4. 3) The Professional Practice Unit track takes place from years 1 to 6 of education. In years 1 and 2 of this track, students of medicine (a total of 80 students) and nursing (a total of 40 students) learn collaboratively, and half of their study time is allocated to workplace placement in a community medicine setting (representing a primary health care setting) [50–52]. During years 3 to 4 of this track, the medical students are assigned to specialized outpatient clinics (representing a secondary health care setting), and in years 5 and 6, the students are assigned to hospitals (representing a tertiary health care setting). Both the outpatient clinics and the hospitals are located in the region of Marília and are affiliated with FAMEMA [53–56]. 4) The Elective Unit track takes place from years 2 to 6 of education and requires students to complete elective internships lasting for 40 days inside or outside the Marília Medical School [50, 51].

The FAMEMA medical programme is in alignment with the Brazilian Unified Health System, which offers free and hierarchical health care at three levels in accordance with the technological complexity involved, i.e., primary, secondary, and tertiary levels of patient care [57]. The government’s actions are intended to serve 100% of the population and primarily focus on primary care (community medicine), which is responsible for promoting good health conditions, preventing disease, treating and controlling the most frequently occurring diseases, promoting health recovery, and/or rehabilitating people. In the Brazilian context, approximately 80% of health demands are met at the level of primary care (community medicine) [57–59].

The placement of FAMEMA students in community medicine is determined by FHS units that are distributed across the neighbourhoods of the city of Marília. These community medicine placements have been part of the FAMEMA curriculum since 2003 [47, 50]. As part of these placements in the FHS units, the students engage in activities to provide community medicine under the supervision of the FHS care team and clinical teachers from FAMEMA. These activities involve home visits, follow-up visits and health projects with a focus on health surveillance[44–46]. Each FHS provides care to 3,000 to 4,000 community inhabitants (750 to 800 families) that live in the surrounding territory. The health care team of an FHS consists of 8 to 15 members from various disciplines, including nursing, medicine (with postgraduate training in various disciplines), dentistry, as well as agents of community health [46]. The latter term refers to staff who have a secondary school degree and who assist in the health promotion actions performed by the FHS [57–59].

The FHS team works in a building with a reception room, medical, nursing, and dental offices, a vaccine room, a pharmacy, a small procedures room, a room for meetings and group care, bathrooms, and a kitchen. However, most activities are performed during visits to the homes of local residents. Thus, part of the team is always away on visits, while the other team members are in the FHS centre. Students complete their FHS placements in groups of 12, which are accompanied by two supervisors (a physician and a nurse, both of whom are teachers at FAMEMA), three times per week for 4 h per visit. In this context, students are integrated into the FHS health team and participate in health care activities for two years. During this period, they perform health care activities to benefit individuals, families and the community.

The supervisor decides which new individual or family a student will see for a home visit and when these visits will occur based on the supervisor’s assessment of the student. During these home visits, the supervisor is accessible in the local area or at the FHS. In addition, she or he accompanies one pair to conduct direct supervision. This student pair generally faces a more complex situation or seems to be more fragile. The other student pairs act without direct in-the-room supervision and can call the supervisor if they experience difficulties to ask for guidance, to ask the supervisor to join the home visit or to have the patient taken to the FHS centre. Each student pair is expected to be able to follow 5 families over the first year in addition to other activities.

Regarding assessment and feedback, the supervisor produces a formative assessment at both the individual and group levels on a daily basis. Each student receives a systematic verbal evaluation when performing health care activities under direct supervision once per week. The frequency of this feedback can be increased if requested by the student or when the supervisor deems it necessary to do so. Every other week, the supervisor evaluates the student´s reflective portfolio. In addition, students engage in discussions with the supervisor regarding all the activities performed by the students,which offers the opportunity for the students to receive new formative assessments and to share the actions they performed with the rest of the FHS team. In addition, a global formative knowledge test is administered once every three months, which includes a remediation plan that is to be followed over subsequent months. The student has two consecutive chances to redo the test (the summative aspect of this knowledge test). Assessment of student performance takes place in the simulation laboratory of FAMEMA at the ends of years 1 and 2 and features the same remediation, number of redos and summative character.

### Delphi study process

Our study was conducted by using a modified, 2-round Delphi process featuring interaction between a writing team and a multiprofessional panel of 15 content experts from FAMEMA. The same members participated in both rounds of the process. The goal of the Delphi study was to define a full set of EPAs representing the authentic participation in community medicine of FAMEMA students during their first and second years of education. The breadth and scope of each EPA was elaborated in the form of a seven-category description in accordance with EPA writing guidelines [2, 44–46]. The EPAs defined will subsequently be embedded in not yet defined entry into postgraduate medical training EPAs for the FAMEMA context.

### Panel selection and writing team

The Delphi panel consisted of purposely selected physicians and nurses from the FAMEMA faculty. The selection criteria included a minimum of ten years of clinical supervision experience, including substantial experience in community medicine and teamwork in the context of the FHS. Another selection criterion was involvement in the continuous curriculum development process at FAMEMA (e.g., individuals who served as course directors or unit coordinators were included). The writing team consisted of the authors of this manuscript (IF, YH and HP), who had combined expertise of the community medicine context at FAMEMA and EPA content elaboration and validation for undergraduate medical education.

### Identification and drafting of EPAs for community medicine in undergraduate medical education

The writing team identified potential EPAs for the year 1 and 2 placements in community medicine at FAMEMA and iteratively elaborated the content of each EPA. The content elaboration included the qualitative analysis and synthesis of written documents that served as the basis for the FAMEMA medical programme, in particular with respect to the placement of medical students in the community. These documents included health policies from the Brazilian Ministry of Health and Ministry of Education [57–59], the National Curriculum Guidelines (NMCG) [49] and the Medical Pedagogical Project of FAMEMA and its medical course notebooks [47], all of which have been used for undergraduate medical education. This step focused on identifying and incorporating the expected knowledge, skills, attitudes and competencies that are required by medical students in their placement in community medicine. The identification and assignment of the appropriate level of supervision for medical students in community medicine during their first and second years of education was based on the supervision levels that were applicable to undergraduate medical education [1]: level 1 - be present and observe, level 2 - act with direct, proactive supervision, i.e., with a supervisor physically present in the room, and level 3 - act with indirect, reactive supervision, i.e., readily available on request. In this process, the experience of one author (IF) as a supervisor for this community medicine setting was helpful in elaborating the most closely aligned content and FAMEMA’s own construction logic for the community medicine EPAs.

The list and descriptions of EPAs were organized into three thematic overarching EPA domains under the auspices of the notion of “integrality of care”, which is commonly used in the Brazilian context. Here, integrality in health refers to the “fundamental principle of public health systems which guarantees citizens the right to be assisted, from disease prevention to the most difficult treatment of a condition, not excluding any disease, prioritizing activities and preventive care services without prejudice. Integrality implies a healthcare and healthcare management that recognizes the autonomy and cultural and social diversity of individuals and populations”[60].

### Questionnaire design and establishment of consensus

The writing team drafted the Delphi study questionnaire, which included the full list of the titles of the proposed EPAs for student participation in community medicine and descriptions of the content of each EPA category. The questionnaire asked the panellists to provide three ratings of the completeness of the description of the EPAs: one rating of the list of EPAs; one rating of the ‘title’, ‘specifications and limitations’, and ‘knowledge, skills and attitude (KSA)’ content sections; and one rating of the ‘conditions and implications of the entrustment decision’. [2]. In each Delphi round, the panellists provided individual ratings in response to statements regarding different EPA aspects on a four-point Likert scale (1 = fully disagree, 2 = partly disagree, 3 = partly agree and 4 = fully agree) as well as narrative comments regarding potential improvements [2]. To ensure that consensus was reached among the panellists, a content validity index (CVI) was derived by calculating the percentage of “partly agree” and “fully agree” ratings (3–4) among all ratings (1–4). The threshold for reaching consensus on each statement in the Delphi study was a mean CVI of all panellist ratings of at least 80% [44]. Each rating item was accompanied by a field for narrative comments. The study questionnaire was designed in Word, and the Word files were distributed to the panellists.

### Panel invitation and briefing

In July 2019, researcher IF invited the panellists to two meetings to introduce the Delphi study. IF introduced the panellists to the goal of the planned study, the concept and definition of EPAs, the scope of and procedure involved in a Delphi study and the task assigned to the panellists. This introduction was followed by an interactive discussion. Before starting the Delphi study, all panellists provided written informed consent to participate.

**Round 1.** The first Delphi round started in July 2019, when the panellists were sent the initial draft of the EPAs for student participation in community medicine as drafted by the writing team. The panellists rated the EPA aspects, provided written comments, and proposed additional EPAs [2, 22]. All panellist responses were received by August 2019. The writing team analysed and summarized both the quantitative ratings and narrative comments. The content and elaboration of the EPAs were adjusted by the writing team via an iterative process. The anonymized results of the first Delphi round were shared with the panellists in September 2019 to allow them to raise questions and discuss them with researcher IF, who represented the writing team.

**Round 2.** The second round started in September 2019. The panellists received the anonymized, summarized panel rating results alongside the refined EPA content descriptions. Changes to the initial draft were highlighted specifically. Again, the panellists could supplement their ratings with narrative comments [2, 22]. All panellist responses were received by October 2019. The writing team analysed and summarized both the quantitative ratings and narrative comments. No further Delphi rounds were conducted, as the threshold for consensus was reached for all EPAs and ratings.

### Finalization of the EPAs and category descriptions

The writing team made final changes to the descriptions of the content of the EPAs for students’ participation in community medicine based on the panel member ratings and comments from Round 2. The EPA categories ‘most relevant domains of competence’ and ‘assessment sources’ were defined by the writing team via an iterative process based on the educational context of FAMEMA. Furthermore, special attention was given to the task of harmonizing the structure, language and wording of the EPA descriptions.

### Data analysis

Descriptive statistics were calculated using IBM SPSS Statistics™ software (version 26, 2019). The panellists’ sociodemographic characteristics are expressed in terms of mean and standard deviation. The relative frequency of panellist ratings and the CVIs calculated for each Delphi round are reported.

## Results

### Panel members and Delphi round response rates

A total of 15 content experts participated in the Delphi procedure, nine of whom were physicians (60%, including five males and four females) and six of whom were nurses (40%, including two males and four females). The mean age of the physicians was 57 ± 12 years, and their mean professional work experience was 27 ± 7 years. The mean age of the nurses was 55 ± 9 years, and their mean professional work experience was 25 ± 7 years of work experience. The response rates for both Delphi rounds were 100% (15 of 15 panellists).

### Identification of EPAs for community medicine

The writing team identified a total of 11 EPAs for the engagement of FAMEMA students in community medicine during their first and second years of education. The supervision level at the end of year 2 was operationalized in terms of the student’s ability to act under reactive supervision, i.e., to perform the EPA autonomously in an effective and safe manner with the supervision readily available on request (supervision level 3) [1] in accordance with current practice at the FAMEMA medical school. The panellist ratings of the completeness of the EPA list, expressed in terms of the CVI, were high in both Delphi rounds for the whole panellist group (round 1: 93%; round 2: 95%), with similar rates of approval for the physician (92%, 94%) and nurse subgroups (96%, 96%). The panellists did not propose any additional EPAs regarding the participation of students in community medicine during their first and second years of education. Minor changes were made and incorporated into the EPA titles following the first Delphi round. Table 1 (left) shows the resulting titles of the 11 EPAs resulting from the Delphi procedure, which have been organized into three overarching thematic EPA domains.

**Table 1:** Titles of the 11 EPAs for students training in community medicine organized in accordance with three overarching EPA domains (left). Mean content validity index (%) indicates the completeness of the combined ‘title’, ‘specifications and limitations’, and ‘knowledge, skills, and attitudes’ category descriptions and the ‘conditions and implications of the entrustment decision’ category description in the first and second Delphi rounds (right). The main results are shown for the whole group of Delphi panellists, while those in parenthesis refer to the physician and nurse subgroups, respectively

No.	EPA domains and EPAs	CVI (%) ‘Title’, ‘specifications and ‘limitations’ and ‘knowledge, skills, and attitudes’		CVI (%) ‘Conditions and implication of the entrustment decision’	
		**Delphi round 1**	**Delphi round 2**	**Delphi round 1**	**Delphi round 2**
**1.**	**Integrality of care for the health needs of the individual in all phases of the life cycle**				
**1.1.**	First consultation to diagnose the health needs of the individual	93% (94%, 92%)	95% (94%, 96%)	88% (86%, 92%)	95% (94%, 96%)
**1.2.**	Development and management of the Individual Therapeutic Project (ITP)	93% (94%, 92%)	92% (92%, 92%)	87% (83%, 92%)	88% (89%, 88%)
**1.3.**	Follow-up consultation on individual health needs	93% (97%, 88%)	95% (94%, 96%)	88% (88%, 88%)	93% (92%, 96%)
**1.4.**	Performance of procedures for individual care in health surveillance	97% (97%, 96%)	95% (94%, 96%)	95% (94%, 96%)	95% (94%, 96%)
**1.5.**	Management of health care support strategies	93% (94%, 92%)	95% (94%, 96%)	95% (92%, 100%)	95% (94%, 96%)
**2.**	**Integrality of care for the health needs of the family**				
**2.1.**	First consultation to diagnose family health needs	92% (92%, 92%),	92% (89%, 96%)	95% (94%, 96%)	90% (86%, 96%)
**2.2.**	Development and management of family health needs	93% (94%, 92%)	95% (94%, 96%)	95% (94%, 96%)	93% (92%, 96%)
**2.3.**	Follow-up consultation on family health needs	97% (94%, 100%)	97% (97%, 96%)	95% (92%, 100%)	93% (92%, 96%)
**3.**	**Integrality of care for the health needs of the community**				
**3.1.**	Diagnosis of health needs in the community	95% (94%, 96%)	95% (92%, 100%)	92% (92%, 92%)	93% (89%, 100%)
**3.2.**	Development and management of the Health Project in the Territory (HPT)	92% (94%, 88%)	95% (92%, 100%)	88% (92%, 83%)	93% (92%, 96%)
**3.3**	Follow-up of the Health Project in the Territory (HPT)	93% (94%, 92%)	93% (92%, 96%)	93% (91%, 96%)	92% (89%, 96%)

### Content elaboration and validation of EPAs for community medicine

Table 1 (right) depicts the panellist ratings of the content descriptions of the 11 individual EPAs for community medicine, once again for the whole group of panellists as well as for the physician and nurse subgroups. In the first Delphi round, the CVI for the ratings of the whole group of panellists was already high, with no relevant differences in the responses between the physician and nurse subgroups. A total of 57 narrative comments offered proposals for changes and clarifications that were used by the writing team to improve the description of the content of the EPAs. The results of the second Delphi round show a high degree of consensus among the panellists concerning the content of the EPAs regarding the participation of students in community medicine during their first and second years of education.

### Resulting EPAs for community medicine

The content elaboration and validation produced two-page descriptions of each EPA. Table 2 presents the full 7-category description of EPA 1.1, ‘First consultation to diagnose the health needs of an individual’, as an example. Table 3 provides an overview of the short summary descriptions of all 11 undergraduate EPAs for community medicine. The full set of all 11 EPAs is provided in the appendix alongside their 7-category descriptions.

**Table 2:** Description of EPA 1.1. as an example of an undergraduate EPA for community medicine

Title: First consultation to diagnose the health needs of the individual (EPA 1.1)
**Short description.** The student, in pairs of two, performs this activity during a home visit in the territory and alternates with student partner in the roles of the executor and the observer. He or she introduces himself/herself to the person (patient), identifies the person, and documents the history of the person’s life, creating a spontaneous account guided by the classical principles of anamnesis and performing a general physical examination of the person with dexterity. Based on the information found and using clinical-epidemiological reasoning, the student develops a diagnosis of the individual’s health needs **Specifications. To perform this EPA, the student**: • Communicates empathetically, respectfully, ethically and effectively with the patient and his or her family. • Performs anamnesis and clinical examination with the patient’s informed consent and builds trust and develops a collaborative relationship. (Home visits must be prioritized to focus on persons diagnosed with diabetes; persons diagnosed with hypertension; pregnant women; children under five years of age; elderly people; and people who are mentally ill, bedridden, physically disabled or carriers of infectious disease). • Uses biosafety standards and follows protocols and service guides. • Evaluates the patient’s general appearance, mental state, vital signs, anthropometric data, mucosa, skin and skin attachments, head and neck, and cardiocirculatory and respiratory systems. Gathers basic diagnostics of abnormalities (e.g., general state, facies, gait, mental orientation, weight, blood pressure and pulse, breath and cardiac frequency, and aspects of nutrition and hydration). • Uses the patient’s clinical history (interview) and the physical examination as aids to develop clinical-epidemiological reasoning and diagnose individual health needs. • Perceives risk factors and the general health condition of the person. In cases of abnormal signs, the student must refer the person to the Family Health Strategy (FHS) unit. In cases of imminent risk, the student must take the patient to the FHS or request help. • Identifies the most common pathologies in the locality/region, perceives their evolution, and ultimately recognizes their most frequent complications. • Based on the literature, provides guidance regarding personal and environmental hygiene (vector control), nutrition (diets to control diabetes, hypertension, and weight reduction), ergonomics, physical activity, sexually transmitted diseases, smoking and alcohol control, adherence to treatment plans, self-medication, access to and use of FHS resources (the women’s health prevention programme; the men’s health prevention programme; and prenatal, puerperium, and child health services). • Based on the literature, answers questions related to health promotion, disease prevention, complications, and risks of the main pathologies present in the locality/region (fever, hypertension, diabetes, dengue, cold, influenza, dehydration, tuberculosis, and the control and transmission of sexually transmitted diseases), smoking and adolescent pregnancy. • Reports home visits to the FHS team, the student group, and the supervisor. • Records relevant information in the patient’s medical record and in the e-SUS AB maintained by the Data Centre of the Brazilian Unified Health System (DATA-SUS) in a clear, organized, and problem-oriented way. **Limitations. This EPA does not apply to/a closer level of supervision is needed in case of the following**: a. Patients in a generally poor condition; terminal patients; haemodynamically unstable patients; and patients with a history of psychiatric illness, a history of violence or drug or alcohol addiction. b. Patients who refuse care either before or during the care. c. Physical examination of newborns, infants, adolescents and pregnant women (conducted alongside the supervisor). d. Specific physical examination and collection of a pap smear (conducted only in the FHS unit). e. In certain risky situations (in the presence of a dangerous animal, drug deal, or gang fight), the student pair must return to the FHS unit. f. If a student is required to work alone, he or she must work only at the FHS
**Conditions and implications of the entrustment decision.** The observations and information collected and compiled by the student form the basis for the expanded diagnosis of the health needs of the patient and the patient care plan without immediate detailed examination by the supervising physician. The patient’s medical history, findings and records should be reviewed by the student during home visits and by the supervisor at the next regular patient home visit or at an appointment at the FHS unit. The supervisor must encourage the student to reflect on the patient’s reality and to share information with the team
**Supervision level at the training stage.** The student should be observed and accompanied over the two years and at the end of the second year: the student is able to perform the EPA under reactive supervision, i.e., to perform the EPA autonomously and in an effective and safe manner with supervision readily available on request (supervision level 3).

**Table 3:** Summary descriptions of the 11 undergraduate EPAs for community medicine

1. Integrality of care for the health needs of the individual in all phases of the life cycle
**EPA 1.1. First consultation to diagnose the health needs of an individual** The student, in pairs of two, performs this activity during a home visit in the territory and alternates with student partner in the roles of the executor and the observer. He or she introduces himself/herself to the person (patient), identifies the person, and documents the history of the person’s life, creating a spontaneous account guided by the classical principles of anamnesis and performing a general physical examination of the person with dexterity. Based on the information found and using clinical-epidemiological reasoning, the student develops a diagnosis of the individual’s health needs **EPA 1.2. Development and management of the Individual Therapeutic Project (ITP)** The student, in pairs of two, discusses the individual health needs diagnosis of the visited person upon his/her return to Family Health Strategy (FHS) unit. There, the two students present the case to the whole group (including the supervisor, health care team and other students), who jointly conduct a provisory ITP based on the principles of health surveillance. This ITP is discussed with the FHS team and finalized with the action plan, and tasks for follow-up are distributed. The students register the home visit and ITP plan in the patient record and with the Data Centre of the Brazilian Unified Health System (DATA-SUS). **EPA 1.3. Follow-up consultation concerning individual health needs** The student, in pairs of two, conducts home visits for follow-up consultations; this activity includes ITP implementation and discussion of the ITP with the patient and his or her family. The patient’s adherence to the ITP and the results are analysed. The student and his or her partner always collect a complementary history and re-examine the patient. They continuously monitor the ITP and are eventually accompanied by the FHS team. Because health needs are likely to change, new visits for follow-up, evaluation and reassessment of the adequacy of the proposed care plan are necessary. **EPA 1.4. Performance of procedures for individual care in health surveillance** The student conducts a defined set of general medical procedures at a primary care level upon patient request. **EPA 1.5. Management of health care support strategies** The student, in pairs of two, implements health care support strategies to provide integral, humanized care and facilitate hierarchical access.
**2. Integrality of care for the health needs of the family**
**EPA 2.1. First consultation to diagnose family health needs** The student, in pairs of two, performs this activity during a home visit in the territory and alternates the roles of executor and observer with his or her student partner. The student is able to conduct home visits autonomously and to collect the family history and perform a general physical examination of the informant family member. He or she collects information regarding family history, interpersonal relations, educational and financial conditions, and social support networks. The student analyses the obtained information and, taking into account the evidence drawn from clinical epidemiologic data, produces a diagnosis of the family’s health needs, choosing priorities and working with the FHS team to improve dimensions of health surveillance. **EPA 2.2. Development and management of family health needs** The student, in pairs of two, performs this activity in the FHS unit. The student and his or her student partner present and discuss all family findings with the health team, make a family health needs diagnosis and, alongside the supervisor, construct a health care plan for the family that takes into account the Programmatic Actions and Care Line mandated for primary care in the municipality. The main types of care are prenatal and puerperal care; child health care until age 5; the national immunization programme; prevention of breast, uterine and prostate cancer; an anti-smoking programme; and the control of diabetes and hypertension. The family health plan is discussed, shared, assessed for adequacy and adjusted in consultation with the family members, and the student is accompanied and assessed during home visits. **EPA 2.3. Follow-up consultation on family health needs** The student, in pairs of two, performs home visits to facilitate follow-up with respect to the family health care plan. He or she performs a complementary clinical history and examination and follows the evolution of the inclusion of the family member in Programmatic Actions and the local Care Lines. The student verifies the implementation of these aspects and discusses them with the patient and his or her family. The student identifies new risky situations and family vulnerabilities. Adherence to the programme and the results are analysed. The student continuously monitors family health care needs and is eventually accompanied by the FHS team. With this interaction and community home visit practices, the student provides information to FHS, thus contributing to the accuracy of primary health care indicator identification.
**3. Integrality of care for the health needs of the community**
**EPA 3.1. Diagnosis of health needs in the community** The student, in pairs of two, diagnoses community health needs via an interpretation of different indicators to serve as a foundation for an appropriate health care plan. Epidemiological data and clinical information related to isolated cases from the territory that forms part of the general district and community knowledge support the evidence-based diagnosis of community health needs. **EPA 3.2. Development and management of the Health Project in the Territory (HPT)** The student, in pairs of two, participates actively in community health planning. He or she discusses the diagnosis of community health needs with the team and the supervisor and subsequently participates in the planning of a broad approach that has a significant community component, which may be developed in the FHS unit or in engagement with some other social body as a Health Project in the Territory - HPT (*Projeto de Saúde no Território - PST*). The student promotes health education activities through the implementation of the Health in School Programme (HSP) (*Programa de Saúde na Escola -PSE*). **EPA 3.3. Follow-up of the Health Project in the Territory (HPT)** The student, in pairs of two, follows the implemented HPT, verifying community adherence to the community health needs care plan and analysing the results for continuous improvement and readjustment. Epidemiological data must be collected, and the HSP programme must be continued. A general overview of the territory must be maintained, and the obtained information must be entered into the DATA-SUS to store it securely.

## Discussion

EPAs have been employed to operationalize competency-based medical education by a steadily increasing number of post- and undergraduate medical training programmes [1–6]. The present study contributes to this worldwide development by expanding the application of the EPA framework to both the start of undergraduate medical education and the context of community medicine. Using a systematic, Delphi-based approach, this study produced a full 7-category description of a total of 11 EPAs for the training and participation of medical students in community medicine during their first and second years of education. In the following, we discuss the process and results of our study in light of the EPA literature and note the study’s specific features pertaining to the context of community medicine.

With regard to the EPA definition process, we employed a modified Delphi study procedure to identify and elaborate undergraduate EPAs in community medicine [44–46]. We purposively chose the Delphi procedure because it allows for the anonymized, nonhierarchical content validation of EPAs and represents an established method of defining EPAs [2, 22]. Two special features of our Delphi study should be emphasized. First, the panel of content experts was selected to be interprofessional in order to reflect the workplace reality of community medicine, in which context medical students are trained and supervised by both physicians and nurses. Other efforts to define undergraduate EPAs have generally involved expert panellists drawn from different medical disciplines but not from other health professions. Second, we combined the ‘title’, ‘specifications and limitations’ and ‘knowledge, skills and attitude’ EPA categories into one item that was rated by the expert panel in the Delphi procedure. We believe that these EPA categories jointly describe the breadth and depth of the relevant EPAs and that this approach allowed valuable time to be saved during the Delphi process, both for the expert panellists and for the writing team. Overall, it required only two Delphi rounds and a few months to reach a consensus regarding the complete description of undergraduate EPAs for community medicine. Several additional factors may have contributed to this efficiency, such as the use of best practice experiences drawn from the literature to guide the process and the establishment of a writing team with expertise both in the definition of EPAs and the content of the envisioned EPAs [2, 8, 22, 27].

With regard to the results of the process, the EPAs defined for the participation of undergraduates in community medicine differ substantially from previously reported EPAs for undergraduate medical education in several ways. First, the proposed undergraduate EPAs for community medicine are embedded in the primary care setting, while previously reported undergraduate EPAs have focused primarily on the secondary-to-tertiary-level health care setting [2, 3, 6–14, 25, 26]. This distinction is reflected by the different scopes of the EPAs in each case, with the proposed community medicine EPAs going beyond primary curative approaches to the care of patients by explicitly including health promotion, disease prevention and rehabilitation. Furthermore, the EPAs for community medicine extend beyond the scope of individual care by including care related to families and the community. Second, the proposed EPAs for community medicine are larger in size and less granular, for instance, by including a full patient consultation under indirect supervision. This characteristic of the community medicine EPAs may reflect the fact that the activities of students participating in community medicine involve primarily low-stakes decisions with little immediate risk, decisions which are often related to lifestyle habits, sanitary conditions and basic care for common diseases that are no longer fully verified or reiterated by professionals after students have qualified for level 3 supervision. This focus of community medicine stands in contrast to the high-stakes, high-risk situations that frequently occur in the tertiary health care setting, for which each step of the patient consultation must be double-checked by a supervising physician before the next step can be taken. Therefore, as illustrated by Chen et al., parts of the patient consultation are nested in smaller, separate EPAs, such as ‘gather information from a medically stable patient with a common chief complaint’ or ‘share information about the patient’s care, including diagnosis and management plan, with a patient in no significant physical or emotional distress’ [28]. Third, community medicine allows for unique workplace-based, interprofessional learning experiences, as these EPAs are carried out in pairs of medical and nursing students. Finally, the EPAs for community medicine are intended for students in their first and second years of medical education, thereby encouraging early participation in real patient care, while previously reported undergraduate medical EPAs are mainly intended for students entering residency or during the final phase of undergraduate medical education [28, 37, 38, 59]. A synthesis of the narrative comments made by the Delphi panellists can be found in a complementary publication [61].

The 11 EPAs defined and reported for community medicine in undergraduate medical education have implications regarding the future development of the field. For FAMEMA in Brazil, these EPAs represent a first step toward a new approach to competency-based medical education. The EPAs used in community medicine will be implemented as overarching outcomes during the first two years in undergraduate medical education; i.e., they will be implemented in the teaching, learning and assessment of students and in the context of faculty development for supervisors and teachers in the programme. Subsequently, EPAs will be defined for the following 4 years of the medical undergraduate programme and the following 2 years of the nursing programme; thereafter, EPAs for the postgraduate education and working phases may be formulated. For other medical schools, especially schools that are involved with community medicine in a manner similar to FAMEMA, this report can provide support and stimulate consideration of the development and implementation of EPAs as part of their curricula.

This work faces certain limitations. The undergraduate EPAs for community medicine discussed here represent the results of a consensus among professionals from one medical school and invited content experts. The generalizability of these results to other medical schools and the contexts in which they operate may be limited, and other groups of content experts may come to different results. In the future, the definition of supervision level 3 would need a clarification of how detailed the findings are being checked by the supervisor and how quickly the supervisor can be present in case urgent help is needed. As wells as the interprofessional nature of working of a medical students in a pair with a nursery student needs further exploration and analysis. Furthermore, the students involved in the programme were not included and could have been an additional source for content validation.

## Conclusion

This study reports the results of the definition of 11 EPAs for the training of medical students in community medicine employing a systematic, Delphi-based approach. It expands the application of the EPA framework to both early undergraduate medical education and to a primary health care setting. This report aims to support other medical schools with training in the primary health care context in the development of EPAs in their curricula.

## Electronic supplementary material

Below is the link to the electronic supplementary material.

Supplementary Material 1

## Data Availability

The dataset used and analysed during this study is available from the corresponding author upon reasonable request. The data cannot be publicly shared based on ethical consideration regarding a potential allocation of data to study participants.

## List of abbreviations

CBME: competency-based medical education.
CVI: content validity index.
EPAs: entrustable professional activities.
FAMEMA: Faculdade de Medicina de Marília (Marilia Medical School).
FAPESP: São Paulo Research Foundation.
FHS: Family Health Strategy.
HP: Harm Peters.
HPT: Health Project in the Territory.
HSP: Health in School Program.
IF: Ieda Francischetti.
ITP: Individual Therapeutic Project.
KSA: knowledge, skills and attitude.
MPP: Medical Pedagogical Project.
NMCG: National Medical Curriculum Guidelines.
PBL: Problem-based Learning.
UME: undergraduate medical education.
UPP: Unit of Professional Practice.
USA: United States of America.
YH: Ylva Holzhausen.
