# Supplementary material for: Entrustable professional activities for Junior Brazilian Medical Students in community medicine
Source: BMC Med Educ. 2022 Oct 25;22:737. doi: 10.1186/s12909-022-03762-4 (PMC9598029; doi:10.1186/s12909-022-03762-4)
Supplement: Supplementary file 1 — Supplementary Material 1 [file 12909_2022_3762_MOESM1_ESM.pdf]

Appendix. EPAs for Community Medicine - EPA Descriptions

EPA Domain 1 - Integrality of care for the health needs of the individual in all phases of the life cycle

**Title**	**1.1 First consultation to diagnose the health needs of the individual (EPA 1.1)**
Short description	The student, in pairs of two, performs this activity during a home visit in the territory and alternates with student partner in the roles of the executor and the observer. He or she introduces himself/herself to the person (patient), identifies the person, and documents the history of the person's life, creating a spontaneous account guided by the classical principles of anamnesis and performing a general physical examination of the person with dexterity. Based on the information found and using clinical-epidemiological reasoning, the student develops a diagnosis of the individual's health needs.
Specifications	**To perform this EPA, the student:**<br>• Communicates empathetically, respectfully, ethically and effectively with the patient and his or her family.<br>• Performs anamnesis and clinical examination with the patient's informed consent and builds trust and develops a collaborative relationship. (Home visits must be prioritized to focus on persons diagnosed with diabetes; persons diagnosed with hypertension; pregnant women; children under five years of age; elderly people; and people who are mentally ill, bedridden, physically disabled or carriers of infectious disease).<br>• Uses biosafety standards and follows protocols and service guides.<br>• Evaluates the patient's general appearance, mental state, vital signs, anthropometric data, mucosa, skin and skin attachments, head and neck, and cardiocirculatory and respiratory systems. Gathers basic diagnostics of abnormalities (e.g., general state, facies, gait, mental orientation, weight, blood pressure and pulse, breath and cardiac frequency, and aspects of nutrition and hydration).<br>• Uses the patient's clinical history (interview) and the physical examination as aids to develop clinical-epidemiological reasoning and diagnose individual health needs.<br>• Perceives risk factors and the general health condition of the person. In cases of abnormal signs, the student must refer the person to the Family Health Strategy (FHS) unit. In cases of imminent risk, the student must take the patient to the FHS or request help.<br>• Identifies the most common pathologies in the locality/region, perceives their evolution, and ultimately recognizes their most frequent complications.<br>• Based on the literature, provides guidance regarding personal and environmental hygiene (vector control), nutrition (diets to control diabetes, hypertension, and weight reduction), ergonomics, physical activity, sexually transmitted diseases, smoking and alcohol control, adherence to treatment plans, self-medication, access to and use of FHS resources (the women's health prevention programme; the men's health prevention programme; and prenatal, puerperium, and child health services).<br>• Based on the literature, answers questions related to health promotion, disease prevention, complications, and risks of the main pathologies present in the locality/region (fever, hypertension, diabetes, dengue, cold, influenza, dehydration, tuberculosis, and the control and transmission of sexually transmitted diseases), smoking and adolescent pregnancy.<br>• Reports home visits to the FHS team, the student group, and the supervisor.<br>• Records relevant information in the patient's medical record and in the e-SUS AB maintained by the Data Centre of the Brazilian Unified Health System (DATA-SUS) in a clear, organized, and problem-oriented way.
Limitations	**This EPA does not apply to/a closer level of supervision is needed in case of the following:**<br>a. Patients in a generally poor condition; terminal patients; haemodynamically unstable patients; and patients with a history of psychiatric illness, a history of violence or drug or alcohol addiction.<br>b. Patients who refuse care either before or during the care.<br>c. Physical examination of newborns, infants, adolescents and pregnant women (conducted alongside the supervisor).<br>d. Specific physical examination and collection of a pap smear (conducted only in the FHS unit).<br>e. In certain risky situations (in the presence of a dangerous animal, drug deal, or gang fight), the student pair must return to the FHS unit.<br>f. If a student is required to work alone, he or she must work only at the FHS unit.
Knowledge, skills and attitude	**Knowledge:**<br>Recognize the care organization (work process/setting); the structure and function of the human body; the presentation and pathophysiology of normal clinical pictures and common first care indicators (inflammation, infection, dehydration, malnutrition, hypertension, alteration of the mental state during the examination, depression); the principles and techniques of communication (verbal and nonverbal); the structure of anamnesis (current complaints; history; personal, family, social and medical history; habits; work conditions; transport; income; intolerances; and questions regarding organs and systems); the principles of hygiene measures and infection prevention; and concepts regarding life quality and health needs; concepts and techniques of differential diagnosis (common headache, upper respiratory infection, dengue, anaemia, dehydration, hypertension, diabetes, obesity, depression, alcoholism, convulsion, and smoking). Know vaccination indications and schedules for healthcare at all life stage, prevention routines, prenatal care and childcare routines.<br>**Skills:**<br>Form an overall impression of the patient; engage in patient-centred discussion; form a trusting professional-patient relationship; collect a structured medical history; perform a structured physical examination, identifying typical and common signs of frequent complaints in first care; recognize relevant normal findings; develop clinical reasoning; synthesize the patient's findings, including the identification of uncertain findings that should be re-examined; account for differential diagnostic considerations; and form a general impression of the house, the patient's way of life, the patient's life satisfaction and the diagnosis.<br>**Attitude:**<br>Open and respectful communication with the partner, supervisor, patient, and family; attention to diversity (gender, age, and culture); use of language that is understandable to patients; compliance with hygiene regulations; respect for the privacy and confidentiality of patient information; responsibility in health practice; commitment to the patient; reflection on the patient encounter (attitudes and feelings) and health needs as well as the expected dimensions of care; and recognition of the importance of the team and that of sharing information with the FHS team.
Conditions/implications of the entrustment decision	The observations and information collected and compiled by the student form the basis for the expanded diagnosis of the health needs of the patient and the patient care plan without immediate detailed examination by the supervising physician.<br>The patient's medical history, findings and records should be reviewed by the student during home visits and by the supervisor at the next regular patient home visit or at an appointment at the FHS unit. The supervisor must encourage the student to reflect on the patient's reality and to share information with the team.
Domain of competence	Health surveillance/attention to health needs: integrality of care ranging from individual to collective needs, including the organization and management of care integrality.
Assessment sources	Reflexive portfolio written on paper concerning practical activities.<br>Teacher's written assessment of student performance in practical activities (4 x per year).
Supervision level at the training stage	The student should be observed and accompanied over the two years and at the end of the second year:<br>The student is able to perform the EPA under reactive supervision, i.e., to perform the EPA autonomously and in an effective and safe manner with supervision readily available on request (supervision level 3).

Title	1.2 Development and management of the Individual Therapeutic Project (ITP) (EPA 1.2)
Short description	The student, in pairs of two, discusses the individual health needs diagnosis of the visited person upon his/her return to Family Health Strategy (FHS) unit. There, the two students present the case to the whole group (including the supervisor, health care team and other students), who jointly conduct a provisory ITP based on the principles of health surveillance. This ITP is discussed with the FHS team and finalized with the action plan, and tasks for follow-up are distributed. The students register the home visit and ITP plan in the patient record and with the Data Centre of the Brazilian Unified Health System (DATA-SUS).
Specifications	**To perform this EPA, the student:**<br>• Communicates empathetically, respectfully, ethically and effectively with the patient and his or her family.<br>• Synthesizes the findings and clearly and empathetically presents the diagnosis of the person's individual health needs to other members of the group, the FHS team and the supervisor.<br>• Offers an integrated perception of the patient, his or her social reality (access to goods and services) and his or her general risk factors.<br>• Designs an integrated, multiprofessional care plan in accordance with the patient's symptoms, findings, clinical picture and attitudes based on ethical principles, moral values, evidence from the literature, the socioeconomic conditions of the person/family in question and available health resources.<br>• Accepts contributions and divergent thoughts and participates productively in the construction of the care plan for the person's health needs in the form of an ITP that takes into account all work already completed by the FHS team.<br>• Registers relevant information in the patient medical record and the e-SUS AB maintained by the DATA-SUS in a clear, organized and problem-oriented way.<br>• Refers the patient to another health care level or service when necessary. Completes the document from the FHS under the guidance of the responsible doctor, who must sign and stamp the document.
Limitations	**This EPA does not apply to/a closer level of supervision is needed:**<br>a. Consultations or specific exams that must be scheduled at or referenced by the FHS unit. In this case, the student can observe.<br>b. Patients who refuse care either before or during the care.
Knowledge, skills and attitude	**Knowledge:**<br>Recognize the care organization (work process/setting); health policy and care hierarchy; processes for referral and counterreferral; concepts regarding life quality and health needs; the principles of health surveillance and clinical epidemiology; the structure and functions of the human body; and the presentation and pathophysiology of typical and common clinical pictures/complaints (inflammation, infection, dehydration, malnutrition, hypertension, alteration of the mental state during the examination, and depression). Know priority criteria for patient referral; the principles of hygiene and infection prevention; and the communication techniques (how to make and receive critical feedback) and principles involved in group collaboration.<br>**Skills:**<br>Form an overall impression of the patient; engage in patient-centred discussion, developing clinical reasoning; present a synthesis of the patient's findings, diagnosis and differential diagnosis to the supervising physician; present a general impression of the house, the patient's way of life and the patient's life satisfaction; discuss and present his or her point of view and arguments; and search the literature for qualified information related to the case.<br>**Attitude:**<br>Open and respectful communication with the partner, supervisor and the FHS team; attention to diversity (gender, age, and culture); compliance with hygiene regulations; caution regarding the risks that are present in the territory; use of an ethical, respectful approach; respect for the privacy and confidentiality of patient information; responsibility in health practice; commitment to the patient; reflection on the patient encounter (attitudes and feelings) and on health needs and the expected dimensions of care; recognition of the importance of the team and that of sharing information with the FHS team; recognition of the prior care provided by the FHS team; development of an embracement approach; and the ability to request and provide help.
Conditions/implications of the entrustment decision	The development of an individual health care plan, i.e., the ITP, requires direct supervision so the supervisor can verify the student's clinical reasoning, health needs diagnosis and the joint construction of the plan. The supervisor must encourage the student to reflect on the patient's reality; to adopt a critical, literature-based view; to comprehend the patient's wishes; to develop clinical reasoning skills; and to share information with the team.
Domain of competence	Health surveillance/attention to health needs: integrality of care ranging from individual to collective needs, including the organization and management of care integrality.
Assessment sources	Reflexive portfolio written on paper concerning practical activities.<br>Teacher's written assessment of student performance in practical activities (4 x per year)
Supervision level at the training stage	The student should be observed and accompanied over the two years and at the end of the second year:<br>The student is able to perform the EPA under reactive supervision, i.e., to perform the EPA autonomously and in an effective and safe manner with supervision readily available on request (supervision level 3).

Title	1.3 Follow-up consultation on individual health needs (EPA 1.3)
Short description	The student, in pairs of two, conducts home visits for follow-up consultations; this activity includes ITP implementation and discussion of the ITP with the patient and his or her family. The patient's adherence to the ITP and the results are analysed. The student and his or her partner always collect a complementary history and re-examine the patient. They continuously monitor the ITP and are eventually accompanied by the FHS team. Because health needs are likely to change, new visits for follow-up, evaluation and reassessment of the adequacy of the proposed care plan are necessary.
Specifications	**To perform this EPA, the student:**<br>• Communicates empathetically, respectfully, ethically and effectively with the patient and his or her family.<br>• Performs anamnesis including an examination of the patient's mental state and a clinical examination with the patient's informed consent and builds trust and a collaborative relationship.<br>• Follows biosafety standards, protocols and service guides.<br>• Evaluates the patient's general appearance, mental state, vital signs, anthropometric data, mucosa, skin and skin attachments, head and neck, and cardiocirculatory and respiratory systems.<br>• Gathers basic diagnostics concerning abnormalities (e.g., general state, facies, gait, mental orientation, weight, blood pressure and pulse, breath and cardiac frequency, and aspects of nutrition and hydration).<br>• Uses the patient's clinical history (interview) and physical examination as aids to develop clinical reasoning, reviews the diagnosis of individual health needs and makes appropriate decisions.<br>• Perceives the person's risk factors and general health condition. Faced with abnormal signs, the student must refer the person to the FHS. within cases of imminent risk, the student must take the patient to the FHS or request help.<br>• Identifies the most common pathologies of the locality/region, perceive its evolution and eventually determines its most frequent complications.<br>• Based on the literature, provides guidance regarding personal and environmental hygiene (vector control), nutrition (diets to control diabetes, hypertension, and weight reduction), ergonomics, physical activity, sexually transmitted diseases, smoking and alcohol control, adherence to treatment plans, self-medication, access to and use of FHS resources (the women's health prevention programme; the men's health prevention programme; and prenatal, puerperium, and child health services).<br>• Based on the literature, answers questions related to health promotion, disease prevention, complications and risks of the main pathologies present in the locality/region (fever, hypertension, diabetes, dengue, cold, influenza, dehydration, tuberculosis, and the control and transmission of sexually transmitted diseases), smoking and adolescent pregnancy.<br>• Explores adherence to the ITP during home visits, including degree of satisfaction, successes and difficulties, side effects, and changes in habits and practices.<br>• Discusses the health care plan and its implementation with the patient or responsible party while taking into account the patient's needs, desires, values, degree of autonomy and adherence.<br>• Supports eventual examination requests related to the diagnostic hypothesis based on epidemiological clinical reasoning.<br>• Reports home visits to the FHS team, the student group and the supervisor. Emphasizes strengths and weaknesses of the implementation of the health care plan and seek alternatives.<br>• Registers relevant information in the patient medical record and the e-SUS AB/DATA-SUS in a clear, organized and problem-oriented way.
Limitations	**This EPA does not apply to/a closer level of supervision is needed:**<br>a. Patients in poor general condition; terminal patients; haemodynamically unstable patients; and patients with a history of psychiatric illness, a history of violence or drug or alcohol addiction.<br>b. Patients who refuse care either before or during the care.<br>c. Physical examination of newborns, infants, adolescents and pregnant women (conducted alongside the supervisor).<br>d. In certain risky situations (in the presence of a dangerous animal, drug deal, or gang fight), the student pair must return to the FHS unit.<br>e. If a student is required to work alone, he or she must work only at the FHS unit.
Knowledge, skills and attitude	**Knowledge:**<br>Recognize the care organization; processes for referral and counterreferral; the structure and functions of the human body; the presentation and pathophysiology of typical and common clinical pictures for the different life cycles/complaints (inflammation, infection, dehydration, malnutrition, hypertension, alteration of the mental state during the examination, and depression); principles and techniques of communication; the structure of an anamnesis; semiological knowledge concerning the most frequent physical and mental alterations; principles of hygiene measures and infection prevention; concepts regarding life quality and health needs; concepts and techniques of differential diagnosis (common headache, upper respiratory infection, dengue, anaemia, dehydration, hypertension, diabetes, obesity, depression, alcoholism, convulsion, and smoking); and the concept of health determinants. Know the criteria for patient referral and counterreferral. Know vaccination indications and schedules for healthcare at all life stages, prevention routines, prenatal care and childcare routines.<br>**Skills:**<br>Form an overall impression of the patient (patient 's way of life and life satisfaction), the ITP progress and the patient's ITP adherence; engage in patient-centred discussion; form a trusting professional-patient relationship; collect a structured medical history; perform structured general and specific physical examinations, identifying typical and common signs and recognition of relevant abnormal findings; develop clinical-epidemiological reasoning; synthesize the patient's findings for the supervisor; reiterate the previous diagnosis of health needs or propose a new diagnosis and priorities; present the ITP implementation progress and ITP adherence and suggest its maintenance or adjustment. Record the results in the patient medical record and demonstrate the ability to search the literature for qualified information related to the case.<br>**Attitude:**<br>Open and respectful communication with the partner, supervisor, patients and FHS team; attention to diversity (gender, age, and culture); use of language that is understandable to the patient; compliance with hygiene regulations; respect for the privacy and confidentiality of patient information; responsibility in health practice; commitment to the patient; reflection on the patient encounter (attitudes and feelings) and on health needs and the expected dimensions of care; recognition of the importance of the team and that of sharing information with the FHS team.
Conditions/implications of the entrustment decision	Observations of the patient's progress and the implementation of the ITP do not demand immediate detailed examination by the supervising physician. The medical history, findings and records and the adequacy of the ITP and any adjustments are reviewed by the student during the home visits and by the supervising physician at the next regular visit to the patient or at an appointment with the patient at the FHS unit. The supervisor should encourage the student to focus on patient adherence to the ITP and to analyse and adjust the care plan when necessary.
Domain of competence	Health surveillance/attention to health needs: integrality of care ranging from individual to collective needs, including the organization and management of care integrality.
Assessment sources	Reflexive portfolio written on paper concerning practical activities.<br>Teacher's written assessment of student performance in practical activities (4 x per year).
Supervision level at the training stage	The student should be observed and accompanied over the two years and at the end of the second year:<br>The student is able to perform the EPA under reactive supervision, i.e., to perform the EPA autonomously and in an effective and safe manner with supervision readily available on request (supervision level 3).

Title	1.4 Performance of procedures for individual care in health surveillance (EPA 1.4)
Short description	The student conducts a defined set of general medical procedures at a primary care level upon patient request.
Specifications	**To perform this EPA, the student:**<br>**Supervision level 3**<br>• Communicates empathetically, respectfully, ethically and effectively with the patient and his or her family.<br>• Explains the nature, duration, expected results, complications, side effects and expectations of the procedures in language that is understandable to the patient.<br>• Performs data collection or procedures with the patient's informed consent and builds trust and a collaborative relationship.<br>• Ensures the legitimacy of performing the exam for the benefit of the patient.<br>• Follows biosafety standards, service flows, protocols and service guides.<br>• Considers the patient's general verbal and nonverbal communication.<br>• Practises reporting. Learns and shares safety lessons.<br>• Perceives risk factors and the general health condition of the person. In cases of abnormal signs, the student requests help.<br>• Collects epidemiological data and administers questionnaires.<br>• Based on the literature, provides guidance regarding the procedure, complication, side effects, the results and next care.<br>• Based on the literature, answers questions related to health promotion, disease prevention, complications and risks of the main pathologies related to the procedure.<br>• Performs an ECG.<br>• Applies or changes bandages.<br>• Performs cardiac massage (during health education training only).<br><br>**Supervision level 2**<br>• Administers subcutaneous or intramuscular injection.<br>• Conducts a pap smear test.<br>• Performs a breast examination.<br>• Performs the Guthrie test.<br>• Administers serums or vaccines.<br>• Writes a prescription, performs exams, and makes referrals for standardized cases.
Limitations	**This EPA does not apply to/ a closer level of supervision is needed:**<br>a. All other invasive procedures should be scheduled at the FHS unit.<br>b. The student should be an observer, especially in cases related to newborns, infants, adolescents and pregnant women.<br>c. Urgent and emergency situations.
Knowledge, skills and attitude	**Knowledge:**<br>Recognize the care organization (work process/setting); the structure (anatomy and its variations) and functions of the human body; the presentation and pathophysiology of the pathology in question (inflammation, infection, dehydration, malnutrition, hypertension, alteration of the mental state during the examination, and depression); the principles and techniques involved in physician–patient communication and verbal and nonverbal communication; ethical principles and patient informed consent; hygiene and infection prevention; biosecurity measures; the concepts, techniques, risks, and side effects of procedures; and necessary patient orientations.<br>**Skills:**<br>Form an overall impression of the patient; conduct patient-centred procedures; form a trusting professional-patient relationship; collect important information related to the case; demonstrate the technical capacity to execute procedures; have prior technical training; recognize normal findings and possible complications; document procedures and register samples; and provide patient guidance based on the literature.<br>**Attitude:**<br>Open and respectful communication; attention to diversity (gender, age, and culture); use of language that is understandable to patients; compliance with hygiene regulations; respect for the privacy and confidentiality of patient information; responsibility in health practice; commitment to the patient; recognition of the student's own limitations and willingness to ask for help; transmission of confidence; reflection on the patient encounter (attitudes and feelings) and on health needs and the expected dimensions of care; and recognition of the importance of the team and that of sharing information with the FHS team.
Conditions/implications of the entrustment decision	Based on the analysis of the indication for the procedure, the supervising physician must evaluate the student's ability in the context of his or her previous knowledge, degree of training and emotional preparation as well as the disposition and collaboration of the patient; the supervising physician must also anticipate the safety of the procedure for all involved. The supervising physician must offer face-to-face support and encourage the student to conduct the procedure or guide him or her towards improvement.
Domain of competence	Health surveillance/attention to health needs: integrality of care ranging from individual to collective needs, including the organization and management of care integrality.
Assessment sources	Reflexive portfolio written on paper concerning practical activities.<br>Teacher's written assessment of student performance in practical activities (4 x per year).
Supervision level at the training stage	The student should be observed and accompanied over the two years and at the end of the second year:<br>The student is able to perform the EPA under reactive supervision, i.e., to perform the EPA autonomously and in an effective and safe manner with supervision readily available on request (supervision level 3).

Title	1.5 Management of health care support strategies (EPA 1.5)
Short description	The student, in pairs of two, implements health care support strategies to provide integral, humanized care and facilitate hierarchical access.
Specifications	**To perform this EPA, the student:**<br>• Makes contact, discusses the patient case and conducts a specialized consultation with the Nuclei of Support to Family Health (NSFH) team.<br>• Makes contact, discusses the patient case and conducts a specialized consultation with the Home Care Service (HCS) team.<br>• Produces a synthesis and performs an oral presentation for the partner, supervisor, FHS, NSFH and/or HCS teams.<br>• Discusses the possibilities envisaged for the ITP with the patient to facilitate shared decision making.<br>• Discusses and plans the most suitable referral flow that is compatible with the patient's needs.<br>• Promotes multiprofessional and/or intersectoral actions to address the patient's health needs.<br>• Refers the patient when necessary to another health care level or service (e.g., specialized ambulatory clinics or hospitals).<br>• Provides updates regarding the patient's status at another health care level.<br>• Receives a counterreferred patient to ensure health care continuity at the primary care level.<br>• Records relevant information in the patient's medical record and the e-SUS AB/DATA-SUS in a clear, organized and problem-oriented way.<br>• Completes the document from the FHS under the guidance of the responsible doctor, who must sign and stamp the document.
Limitations	**This EPA does not apply to/ a closer level of supervision is needed:**<br>a. Patients in a generally poor condition; terminal patients; haemodynamically unstable patients; and patients with a history of psychiatric illness, a history of violence and drug or alcohol addiction.<br>b. Patients who refuse care either before or during the care.<br>c. Newborns, infants, adolescents and pregnant women (conducted alongside the supervisor).<br>d. Urgent and emergency situations.
Knowledge, skills and attitude	**Knowledge:**<br>Recognize the care organization (work process/setting) and processes for referral and counterreferral. Know about the FHS and the territory; local risk factors and patient vulnerabilities; and the principles and techniques involved in physician–patient communication, verbal and nonverbal communication, and communication with patients/relatives and the multiprofessional team. Create clear and organized documentation of complete anamneses, physical exams, findings, labour results, the discussed diagnosis of the patient health needs and the ITP. Know concepts regarding life quality and health needs.<br>**Skills:**<br>Form an overall impression of the patient; engage in patient-centred discussion; form a trusting professional-patient relationship; collect a structured medical history; perform structured general and specific physical examinations, identifying typical and common signs of illness; recognize relevant findings; propose a diagnosis; present and discuss the patient case with the partner, the supervisor, and multiprofessional teams; record the results in the patient's medical record; search the literature for qualified information related to the case; sensitize others to resolve and/or help with ITP implementation; and determine when it is necessary to refer the patient elsewhere.<br>**Attitude:**<br>Open and respectful communication; attention to diversity (gender, age, and culture); use of language that is understandable to patients; compliance with hygiene regulations; respect for the privacy and confidentiality of patient information; responsibility in health practice; commitment to the patient; reflection on the patient encounter (attitudes and feelings) and on health needs and the expected dimensions of care; recognition of the importance of the team and that of sharing information with all involved professionals.
Conditions/implications of the entrustment decision	Understanding the health system as a whole is very important for the effective functioning of a physician. When the student identifies the need to adopt a broader view of the patient's health needs and engages in more complex actions at other levels of attention, he or she improves the system's response and his or her own professional intervention capacity. The supervising physician should encourage the student to be critical, to adopt an integral view of the patient case, to be emphatic, to involve others health professionals in discussions and network building to create new possibilities for referral, to follow up with his or her patient when the patient is transferred to another level of health care, and to receive and follow up with the patient in upon counterreferral.
Domain of competence	Health surveillance/attention to health needs: integrality of care ranging from individual to collective needs, including the organization and management of care integrality.
Assessment sources	Reflexive portfolio written on paper concerning practical activities.<br>Teacher's written assessment of student performance in practical activities (4 x per year).
Supervision level at the training stage	The student should be observed and accompanied over the two years and at the end of the second year:<br>The student is able to perform the EPA under reactive supervision, i.e., to perform the EPA autonomously and in an effective and safe manner with supervision readily available on request (supervision level 3).

EPA Domain 2 - Integrality of care for the health needs of the family

Title	2.1 First consultation to diagnose family health needs (2.1)
Short description	The student, in pairs of two, performs this activity during a home visit in the territory and alternates the roles of executor and observer with his or her student partner. The student is able to conduct home visits autonomously and to collect the family history and perform a general physical examination of the informant family member. He or she collects information regarding family history, interpersonal relations, educational and financial conditions, and social support networks. The student analyses the obtained information and, taking into account the evidence drawn from clinical epidemiologic data, produces a diagnosis of the family's health needs, choosing priorities and working with the FHS team to improve dimensions of health surveillance.
Specifications	**To perform this EPA, the student:**<br>• Communicates empathetically, respectfully, and ethically and exhibits a commitment to the family's quality of life.<br>• Diagnoses basic abnormalities (family members' health conditions, house-related and environmental conditions, basic sanitation, local security, consumption capacity, and access to services).<br>• Recognizes family dynamics and bonding beyond the clinical history of the person.<br>• Uses diagrams or genograms, ecomaps and social network references to understand the family and its health needs in greater detail. Considers the social network and possibilities of caregivers.<br>• Verifies and interprets the living conditions of the family in relation to community health.<br>• Identifies chronic diseases (diabetes, hypertension) and infectious diseases, including disease vectors.<br>• Identifies the vaccination statuses and access to dental care of family members.<br>• Identifies local collective health problems and gathers indicators of life quality.<br>• Identifies the need for family planning.<br>• Teaches environmental health care.<br>• Associates the information collected with local clinical epidemiological evidence to produce a diagnosis concerning family health.<br>• Develops amplified clinical reasoning.<br>• Discusses the collected information with the FHS team and the supervisor.<br>• Records all collected information in the patient and family medical records in a clear, organized and problem-oriented way and registers the data with the e-SUS AB/DATA-SUS (with notifications when necessary). Accesses epidemiological data pertaining to the locality/region and provides this information to other team members.<br>• Provides orientations and clarifies doubts related to health education. Based on the literature, provides guidance and/or answers questions related to health promotion, injury prevention, treatment, complications, and risks of the main pathologies of the locality/region.
Limitations	**This EPA does not apply to/ a closer level of supervision is needed:**<br>a. Patients in poor general condition; terminal patients; haemodynamically unstable patients; and patients with history of psychiatric illness (e.g., schizophrenia, hoarding disorder, or emotional extortion signs), a history of violence or drug or alcohol addiction.<br>b. Newborns, infants, adolescents and pregnant women, specific physical examinations (conducted alongside the supervisor).<br>c. In certain risky situations (in the presence of a dangerous animal, drug deal, or gang fight), the student pair must return to the FHS unit.<br>d. If a student is required to work alone, he or she must work only at the FHS unit.
Knowledge, skills and attitude	**Knowledge:**<br>Recognize the care organization (work process/setting, Programmatic Actions and Health Care Network), health policy and care hierarchy; the principles of health quality, health needs, health surveillance and clinical epidemiology; and community health instruments for the identification of infectious diseases (differentiate cases of compulsory notification), disease vectors and breeding grounds, chronic diseases (risk factors and control measures). Know the vaccination indications and schedules for healthcare at all life stages. Comprehend the relevance of and understand indications suggesting dental care; family planning measures and indications; and environmental health care and health management principles. Comprehend the structure and functions of the human body; the presentation and pathophysiology of typical and common clinical pictures/complaints; the principles and techniques of physician–patient/informant communication and family and teamwork communication; the structure of anamnesis and physical examination; instruments such as diagrams, genograms, ecomaps and social network frameworks; the concepts of the nuclear family and its organization; hygiene and infection prevention; and the concepts and techniques involved in differential family health needs diagnosis.<br>**Skills:**<br>Form an overall impression of the patient and family; engage in family-centred discussion, form a trusting professional-patient relationship; collect a structured medical history; perform a structured physical examination; apply social data collection instruments; recognize the family's way of life; recognize relevant normal and abnormal findings; present a general impression of the house and the family dynamic, interpersonal and social relations, financial and educational support, as well as considerations regarding weaknesses and differential diagnosis, to the supervising physician for discussion; register information concerning the interviewed member in the family medical record and at the e-SUS AB/DATA-SUS (when applicable) and search the literature for qualified information related to the case.<br>**Attitude:**<br>Open and respectful communication; attention to diversity (gender, age, and culture); use of language that is understandable to the interlocutor; caution regarding biosafety and ergonomic measures; use of an ethical, respectful approach that includes a suitable location for examinations, reasonable time management and respect for the privacy and confidentiality of information; responsibility in health practice; commitment to the informant/patient and his or her family; reflection on the patient encounter (attitudes and feelings), the family health needs and the expected dimensions of care; and recognition of the importance of the team and that of sharing information with the FHS team.
Conditions/implications of the entrustment decision	The observations and information collected and compiled by the student form the basis for an expanded diagnosis of family health needs and a broad comprehension of general health community diagnosis, health planning and action implementation. Each step will be discussed with FHS team and the supervising physician.<br>The family context and the family member should be reviewed by the student during the home visits and by the supervising physician when requested at the home visit or at an appointment at the FHS unit. The supervisor should encourage the student to reflect on the family's reality, to comprehend the consequences of the family context and way of life for the individual's health situation, to develop clinical reasoning and the ability to diagnose the family's health care needs and priorities, considering health surveillance data and discussing and sharing information with the health team.
Domain of competence	Health surveillance/attention to health needs: integrality of care ranging from individual to collective needs, including the organization and management of care integrality.
Assessment sources	Reflexive portfolio written on paper concerning practical activities.<br>Teacher's written assessment of student performance in practical activities (4 x per year).
Supervision level at the training stage	The student should be observed and accompanied over the two years and at the end of the second year:<br>The student is able to perform the EPA under reactive supervision, i.e., to perform the EPA autonomously and in an effective and safe manner with the supervision readily available on request (supervision level 3).

Title	2.2 Development and management of family health needs (2.2)
Short description	The student, in pairs of two, performs this activity in the FHS unit. The student and his or her student partner present and discuss all family findings with the health team, make a family health needs diagnosis and, alongside the supervisor, construct a health care plan for the family that takes into account the Programmatic Actions and Care Line mandated for primary care in the municipality. The main types of care are prenatal and puerperal care; child health care until age 5; the national immunization programme; prevention of breast, uterine and prostate cancer; an anti-smoking programme; and the control of diabetes and hypertension. The family health plan is discussed, shared, assessed for adequacy and adjusted in consultation with the family members, and the student is accompanied and assessed during home visits.
Specifications	**To perform this EPA, the student:**<br>• Develops and trains in safety practices.<br>• Practises caution regarding integrated vector control.<br>• Addresses family planning.<br>• Engages with and promotes indicators of quality of life.<br>• Administers and organizes health education practice.<br>• Employs embracement practice.<br>• Works according to the care organization in question (work process/setting/policy).<br>• Communicates empathetically, respectfully, ethically and exhibits a commitment to the family's quality of life.<br>• Performs actions necessary to meet the health needs of the family, including the involvement of family members and the social support network and efficient resource utilization.<br>• Works in accordance with public health policies and the priority actions planned for the locality/region in primary health care.<br>• Engages in joint planning with the FHS, NSFH, and HCS teams and seeks intersectoral actions.<br>• Includes the informant and his or her family in the action programmes and campaigns to promote quality of life, prevention of damage, adherence to treatments and follow-ups with the FHS unit.<br>• Analyses and provides plans and measures; provides specific care in accordance with identified family health needs.<br>• Engages in permanent readjustment to involve family members in the follow-up for the health care plan.<br>• Assesses the evolution of the health care plan over time and rediscusses it.<br>• Develops actions to improve family reception of and inclusion in the health care plan.
Limitations	**This EPA does not apply to/ a closer level of supervision is needed:**<br>**a.** Consultations or specific exams that must be scheduled at the FHS unit or a referral institution. In this case, the student can observe.
Knowledge, skills and attitude	**Knowledge:**<br>Recognize the care organization (work process/setting); health policy; processes for referral and counterreferral; Programmatic Actions and Health Care Network mandated for primary care in the municipality and the care hierarchy; communication principles and techniques; the principles of health surveillance; the principles of clinical epidemiology and risk factors; the structure and functions of the human body; the presentation and pathophysiology of typical and common clinical pictures/complaints; concepts regarding life quality and health needs; concepts regarding group collaboration; and the management of epidemiological data.<br>**Skills:**<br>Form an overall impression of the patient/informant; engage in family-centred discussion; develop a trusting professional-patient relationship; collect adequate information to assess family inclusion in and adherence to the family health care plan; presentation of a synthesis of the general impression of the house, family life quality, family dynamic and signs of abnormalities or uncertain findings for discussion with the supervising physician; propose a way to continue the development of health care plan; register the results in the patient and family medical record; and search the literature for qualified information relevant to the case.<br>**Attitude:**<br>Open and respectful communication; attention to diversity (gender, age, and culture); use of language that is understandable to the interlocutor; compliance with hygiene regulations; caution regarding risks that are present in the territory; use of biosafety and ergonomic measures; use of an ethical, respectful approach including a suitable location for examinations, reasonable time management and respect for the privacy and confidentiality of information; responsibility in health practice; commitment to the family; reflection on the family member encounter (attitudes and feelings) and on the family health needs and evolution of the care plan; recognition of the importance of the team and that of sharing information with the FHS, NSFH and HCS teams; and development of an embracement approach.
Conditions/implications of the entrustment decision	The observations of the family care plan implementation and the inclusion of family members in the Programmatic Actions and Care Line mandated for primary care in the municipality is very important as a health management practice in the community health care area.<br>The results of the use of the plan and its adequacy and readjustments should be reviewed and revised by the students during home visits and by the supervising physician at the next regular visit to the family or at an appointment for the family member at the FHS unit. The supervisor should encourage the student to follow and assess the plan implementation, the family member's inclusion in the plan, the results, and limitations to continuous adjustment based on the family's health needs.
Domain of competence	Health surveillance/attention to health needs: integrality of care ranging from individual to collective needs, including the organization and management of care integrality.
Assessment sources	Reflexive portfolio written on paper concerning practical activities.<br>Teacher's written assessment of student performance in practical activities (4 x per year).
Supervision level at the training stage	The student should be observed and accompanied over the two years and at the end of the second year:<br>The student is able to perform the EPA under reactive supervision, i.e., to perform the EPA autonomously and in an effective and safe manner with supervision readily available on request (supervision level 3).

Title	2.3 Follow-up consultation on family health needs (2.3)
Short description	The student, in pairs of two, performs home visits to facilitate follow-up with respect to the family health care plan. He or she performs a complementary clinical history and examination and follows the evolution of the inclusion of the family member in Programmatic Actions and the local Care Lines. The student verifies the implementation of these aspects and discusses them with the patient and his or her family. The student identifies new risky situations and family vulnerabilities. Adherence to the programme and the results are analysed. The student continuously monitors family health care needs and is eventually accompanied by the FHS team. With this interaction and community home visit practices, the student provides information to FHS, thus contributing to the accuracy of primary health care indicator identification.
Specifications	**To perform this EPA, the student:**<br>• Visits the territory, i.e., the geographic area of action of the FHS team, and recognizes its physical space, environmental protection and risk factors, sociodemographic profile, educational health characteristics of the community and social resources.<br>• Communicates empathetically, respectfully, ethically and effectively with the patient/family/member.<br>• Performs anamnesis, clinical examination and data collection (by administering forms) with the patient's informed consent and build trust and a collaborative relationship.<br>• Follows biosafety standards, protocols and service guides.<br>• Evaluates the patient's general appearance, mental state, vital signs, anthropometric data, mucosa, skin and skin attachments, head and neck, and cardiocirculatory and respiratory systems.<br>• Gathers basic diagnostics concerning abnormalities (e.g., general state, facies, gait, mental orientation, weight, blood pressure and pulse, breath and cardiac frequency, nutrition, and hydration aspects).<br>• Uses the patient's clinical history (interview) and physical examination as aids to develop clinical reasoning, reviews the diagnoses of the family health needs and chooses a priority.<br>• Perceives the risk factors and general health condition of the family. In a vulnerable situation, the student should refer the patient/family to the FHS and promote interprofessional/intersectoral actions. In cases of imminent risk, the student must take the patient to the FHS or request help.<br>• Explores adherence to ITP, Programmatic Actions and the local Care Lines in terms of the degree of satisfaction, successes, difficulties, side effects, and changes in habits and practices during home visits.<br>• Develops a family health needs diagnosis and discusses the actual family health plan, seeking more effective possibilities.<br>• Uses indicators of quality of life and epidemiological surveillance data.<br>• Develops actions to improve family reception of and inclusion in the health care plan.<br>• Based on the literature, provides guidance regarding personal and environmental hygiene (vector control), health education, nutrition (diets to control diabetes, hypertension, and weight reduction), ergonomics, physical activity, sexually transmitted diseases, immunization, guidance for smoking and alcohol control, adherence to treatment, self-medication, access to and use of FHS resources (the women's health prevention programme; the men's health prevention programme; and prenatal, puerperium, and child health services) as well as those of NSFH and HCS.<br>• Based on the literature, answers questions related to health promotion, disease prevention, complications and risks of the main pathologies present in the locality/region (fever, hypertension, diabetes, dengue, cold, influenza, dehydration, tuberculosis, the control and transmission of sexually transmitted diseases), smoking and adolescent pregnancy.<br>• Discusses the family health care plan and its implementation with the family member or responsible party while taking into account the family's needs, desires, values, degree of autonomy, and adherence.<br>• Practises reporting the home visit to the FHS team, student group and supervisor. When necessary, reports to the NSFH and HCS teams. Is able to emphasize strengths and weaknesses of the implementation of the family health care plan, discusses and seek alternatives.<br>• Records relevant information in the patient and family medical record and e-SUs AB by DATA-SUS in a clear, organized and problem-oriented way.
Limitations	**This EPA does not apply to/ a closer level of supervision is needed:**<br>a. Patients in poor general condition; terminal patients; haemodynamically unstable patients; patients with a history of psychiatric illness, a history of violence and drug or alcohol addiction.<br>b. Physical examination of newborns, infants, adolescents and pregnant women.<br>c. In some risky situations (in the presence of a dangerous animal, drug deal, or gang fight), the student pair must return to the FHS unit.<br>d. If a student is required to work alone, he or she must work only at the FHS unit.
Knowledge, skills and attitude	**Knowledge:**<br>Recognize the care organization; health policy, health programs and care hierarchy; processes for referral and counterreferral; the principles of health surveillance, clinical epidemiology and epidemiological surveillance; evidence-based health care; the principles and techniques of communication and group collaboration; the structure and functions of the human body; the presentation and pathophysiology of typical and common clinical pictures for various life cycles/complaints (inflammation, infection, dehydration, malnutrition, hypertension, alteration of the mental state during the examination, and depression); the structure of anamnesis; semiological knowledge regarding the most frequent physical and mental alterations; biosecurity measures and the principles of hygiene and measures for infection prevention; concepts regarding qualify of life and health needs; the concepts and techniques involved in differential diagnosis (common headache, upper respiratory infection, dengue, anaemia, dehydration, hypertension, diabetes, obesity, depression, alcoholism, convulsion, and smoking). Have concepts of chronic and infectious diseases (differentiate cases of compulsory notification). Recognize disease vectors and breeding grounds. Know vaccination indications and schedules for healthcare at all life stages, prevention routines, prenatal care, and childcare routines. Understand the Programmatic Actions and Health Care Network, the current thematic networks mandated for primary care in the municipality and data documentation and index calculation.<br>**Skills:**<br>Form an overall impression of family relations (family members' way of life and satisfaction), the ITP and family health care plan progress and the family members' adherence; engage in patient-centred discussion; form a trusting professional-patient relationship; collect a structured medical history; perform structured general and specific physical examinations, identifying typical and common signs, relevant abnormal findings and collective patterns; develop clinical reasoning and present a synthesis of the findings on the family members and the family case to the supervisor and health teams, including the principal evidence; reiterate the previous diagnosis of family health needs or propose a new diagnosis, adjusting the diagnosis and/or identifying a priority; record the findings in the family member record or at the e-SUS AB by DATA-SUS; search the literature for qualified information related to the case or area health profile; and demonstrate the capability to guide health education activities.<br>**Attitude:**<br>Caution regarding risks present in the territory; responsibility in health practice; development of an embracement approach; open and respectful communication with the partner, supervisor, family/members and health teams; attention to diversity (gender, age, and culture); use of language that is understandable to the interlocutor; use of an ethical, respectful approach including a suitable location for examinations, reasonable time management and respect for the privacy and confidentiality of information; compliance with hygiene regulations; commitment to the patient/family/member; reflection on the patient, family/member and work team encounter (attitudes and feelings) and on health needs, the expected dimensions of care and the health plan progress; and recognition of the importance of the health teams and that of sharing information with the health teams.
Conditions/implications of the	The activities performed and information collected and compiled by the students represent a large part of the primary health care conducted in the territory and serve as the basis for further care for the health needs of the family/community and the treatment care plan. An immediate detailed examination by the supervising physician is unnecessary.

entrustment decision	The patient/family should be approached mostly through continuous home visits. The findings should be recorded adequately and discussed with the health team and supervisor. A diagnosis and care plan should be proposed, and the care plan implementation and results should be assessed and adjusted. The supervising physician should participate and review all student interactions and results. The supervisor must encourage the student to participate in health team activities (home visits, health education, management, and organization), to reflect on the family's reality, to generate perceptions of the territory and the community and to develop epidemiological and clinical reasoning on which the student's actions in the relevant health care area can be based.
Domain of competence	Health surveillance/attention to health needs: integrality of care ranging from individual to collective needs, including the organization and management of care integrality.
Assessment sources	Reflexive portfolio written on paper concerning practical activities.<br>Teacher's written assessment of student performance in practical activities (4 x per year).
Supervision level at the training stage	The student should be observed and accompanied over the two years and at the end of the second year:<br>The student is able to perform the EPA under reactive supervision, i.e., to perform the EPA autonomously and in an effective and safe manner with supervision readily available on request (supervision level 3).

EPA Domain 3 - Integrality of care for the health needs of the community

**Title**	**3.1 Diagnosis of health needs in the community**
Short description	The student, in pairs of two, diagnoses community health needs via an interpretation of different indicators to serve as a foundation for an appropriate health care plan. Epidemiological data and clinical information related to isolated cases from the territory that forms part of the general district and community knowledge support the evidence-based diagnosis of community health needs.
Specifications	**To perform this EPA, the student:**<br>• Visits the territory, i.e., the geographic area of action of the FHS team, and recognize its physical space, environmental protection and risk factors, sociodemographic profile, the educational e-health characteristics of the community and social resources (schools, kindergartens, churches, and community centres).<br>• Uses embracement practice; communicates empathetically, respectfully, and ethically; and is committed to the quality of community life and integration.<br>• Collects information concerning the territory via interviews, anamnesis, clinical examinations, and meetings with community members.<br>• Maps the area (epidemiological maps).<br>• Obtains general data pertaining to the territory from the e-SUS AB by DATA-SUS or other official databases.<br>• Performs an active search and identifies diseases and aggravations with compulsory notifications; records families with low levels of social resources and individuals with chronic diseases.<br>• Participates in community council meetings.<br>• Identifies health risk factors and vulnerabilities in the territory.<br>• Uses indicators, such as birth rate, infant mortality index, human development index, adolescent pregnancy rate, life expectancy of the local community, local unemployment rate and literacy rate, to produce a community health needs diagnosis.<br>• Follows and promotes indicators of quality of life. Supplies information to the e-SUS AB by DATA-SUS.<br>• Exercises caution with respect to biosafety and trains in safety practices.<br>• Works as a team with the student partner, the supervisor, the FSH team, the NSFH team, the HCS team, and people from the community to understand the dynamics of the locality/region (including contiguous rural areas) and local health demands in greater detail.<br>• Discusses the general findings regarding the community and participates in and contributes to the FSH, NSFH, and HCS teams to understand the community health needs and the Unified Health System-UHS (*Sistema Único de Saúde-SUS*) policies and possibilities in greater detail.<br>• Analyses community health needs by way of various indicators to develop an evidence-based diagnosis of community health needs.<br>• Discusses his or her ideas with the student partner, the health team, and the supervisor and discards differential diagnoses.<br>• Develops a health diagnosis for the territory that takes into account the epidemiological surveillance data with his or her student partner, the supervisor and the health team.
Limitations	**This EPA does not apply to/ a closer level of supervision is needed:**<br>a. Community members (patients) who do not have a legal age of consent (children, adolescents), people with physical or mental disability or those who are not willing to interact.<br>b. In some risky situations (in the presence of a dangerous animal, drug deal, or gang fight), the student pair must return to the FHS unit.<br>c. If a student is required to work alone, he or she must work only at the FHS unit.
Knowledge, skills and attitude	**Knowledge:**<br>Recognize the care organization (work process/setting), health policy and care hierarchy; processes for referral and counterreferral; principles of health surveillance, clinical epidemiology and epidemiological surveillance; the principles and techniques of communication and group collaboration; concepts regarding life quality and health needs; evidence-based health care; and data documentation and index calculation. Comprehend the structure and functions of the human body; the presentation and pathophysiology of typical and common clinical pictures/complaints; and concepts related to chronic and infectious diseases (differentiate cases of compulsory and noncompulsory notification). Comprehend the importance of the diagnosis for the adequacy of health care plan. Understand the Programmatic Actions and Care Line mandate for primary care in the municipality. Recognize disease vectors and breeding grounds. Know about biosecurity measures and hygiene standards.<br>**Skills:**<br>Form an overall impression of the community, social resources and territory; engage in community-centred discussion; form a trusting professional-community member relationship; recognize collective patterns and relevant abnormal findings; identify evidence and discard differential diagnoses; present a synthesis of the principal findings and evidence to the supervising physician and the health team; register the findings in the community member record or at the e-SUS AB/DATA-SUS; and search the literature for qualified information related to the area health profile.<br>**Attitude:**<br>Open and respectful communication; attention to diversity (gender, age, and culture); use of language that is understandable to patients; compliance with hygiene regulations; exercise of caution regarding risks that are present in the territory; use of biosafety and ergonomic measures; use of an ethical, respectful approach that includes a suitable location for examinations, reasonable time management and respect for the privacy and confidentiality of information; responsibility in health practice; commitment to the community/community member; reflection on the patient, community and work team encounters (attitudes and feelings), on health needs and on the expected dimensions of care; recognition of the importance of the team and that of sharing information with the FHS team; involvement and communication with patients and the community; and development of an embracement approach.
Conditions/implications of the entrustment decision	The activities performed and information collected and compiled by the students represent a large part of the primary health care in the territory and form the basis for an expanded diagnosis of the health needs of the community/community member and the treatment care plan. An immediate detailed examination by the supervising physician is unnecessary. Diagnosis of community health needs should be proposed for future plan implementation. The supervisor should encourage the student, taking into account the relevant health surveillance data, to reflect on the community reality and to use these instruments to develop clinical reasoning and the first diagnosis and care plan idea on his or her own. Otherwise, the supervisor must encourage the students to share information and discuss it with the team.
Domain of competence	Health surveillance/attention to health needs: integrality of care ranging from individual to collective needs, including the organization and management of care integrality.
Assessment sources	Reflexive portfolio on paper concerning practical activities.<br>Teacher's written assessment of student performance in practical activities (4 x per year).
Supervision level at the training stage	The student should be observed and accompanied over the two years and at the end of the second year:<br>The student is able to perform the EPA under reactive supervision, i.e., to perform the EPA autonomously and in an effective and safe manner with supervision readily available on request (supervision level 3).

Title	3.2 Development and management of the Health Project in the Territory (HPT)
Short description	The student, in pairs of two, participates actively in community health planning. He or she discusses the diagnosis of community health needs with the team and the supervisor and subsequently participates in the planning of a broad approach that has a significant community component, which may be developed in the FHS unit or in engagement with some other social body as a Health Project in the Territory - HPT (*Projeto de Saúde no Território - PST*). The student promotes health education activities through the implementation of the Health in School Programme (HSP) (*Programa de Saúde na Escola -PSE*).
Specifications	**To perform this EPA, the student:**<br>• Knows the territory, i.e., the geographic area of action of the FHS team, and recognize its physical space, environmental protection and risk factors, sociodemographic profile, the educational e-health characteristics of the community and social resources.<br>• Communicates empathetically, respectfully, ethically and effectively with the patient and his or her family.<br>• Follows biosafety standards and service flows, protocols and service guides.<br>• Works together with the partner, the supervisor; and the FHS, NSFH, and HCS teams.<br>• Designs an integrated HTP involving multiprofessional and/or intersectoral actions, including a physical activities programme, community garden, animal castration, intersectoral actions (literacy classes, transportation lines, waste collection, and lighting, security services, among others); determines the necessity of special campaigns (clean city, vaccination, breastfeeding, anti-tobacco, and cancer prevention campaigns); or performs an active search of cases, contaminants, and primary vectors.<br>• Participates in campaigns.<br>• Prioritizes actions planned for the locality/region in the context of primary health care, including promoting immunization practices, facilitating family planning, educating and protecting against domestic violence, providing sexuality education for adolescents, guiding environmental health care measures, exercising caution regarding integrated vector control, identifying infectious and chronic diseases and determining the locations of collective health problems.<br>• Performs actions aimed at fulfilling the HTP with the involvement of the community members or representatives and the social support network with efficient resource utilization.<br>• Plans and develops various health educations activities, promotes leadership and works with community leaders.<br>• Develops participatory health planning steps alongside the FHS team.<br>• Implements an HSP. Promotes adolescent health care education and leadership.<br>• Develops actions to improve family reception and inclusion (participation in decision making).<br>• Registers HTP, HSP and other information in the FHS record and e-SUS AB/DATA-SUS in a clear, organized and problem-oriented way.
Limitations	**This EPA does not apply to / a closer level of supervision is needed:**<br>a. Low consistency of evidence.<br>b. Unavailability of the e-SUS AB/DATA-SUS.<br>c. Inability to ensure follow-up.<br>d. Insufficient information in the patient or family medical record.
Knowledge, skills and attitude	**Knowledge:**<br>Recognize the care organization (work process/setting), health policy and care hierarchy; processes for referral and counterreferral; the principles of health surveillance, clinical epidemiology and epidemiological surveillance; the various types of health planning and the stages of participatory health planning; concepts regarding life quality and health needs; evidence-based health care; data documentation and index calculation; and data registration and interpretation. Understand the Programmatic Actions and Care Line mandated for primary care in the municipality. Comprehend the importance of the health care plan management. Know how to conduct clinical-epidemiological reasoning and follow data pertaining to sentinel events. Know about processes for referral and counterreferral and mortality, morbidity, etc. Recognize the principles and techniques involved in communication and group collaboration.<br>**Skills:**<br>Form an overall impression of the community and community/members and compare it with the data; engage in community-centred discussion; form a trusting professional-community/member and professional–team relationship; plan and develop a community health care approach based on evidence of the community health needs through teamwork; guide health education activities; register data in the community member record or at the e-SUS AB/DATA-SUS; and search the literature for qualified information related to the area health profile or to sentinel cases.<br>**Attitude:**<br>Open and respectful communication; attention to diversity (gender, age, and culture); appropriate use of language; ethical, respectful approach and management of the data and respect for the privacy and confidentiality of patient information; responsibility in health practice; commitment to the community/community member; reflection on the patient, community and work team encounters (attitudes and feelings), on health needs and the expected dimension of care; recognition of the importance of the team and that of sharing information with the FHS team; involvement and communication with patients and the community; and development of an embracement approach.
Conditions/implications of the entrustment decision	The involvement of the student with the community, his or her precise perception of the reality of the community and review of the clinical-epidemiological data are fundamental for the implementation and management of an integrated HTP and HSP. The supervisor should encourage the student, taking into account health surveillance data, to reflect on the reality of the community and, using evidence-based information, to interact with the health team and participate actively in HTP and HSP actions. On the other hand, the supervisor must encourage the student to include community members in decision making.
Domain of competence	Health surveillance/attention to health needs: integrality of care ranging from individual to collective needs, including the organization and management of care integrality.
Assessment sources	Reflexive portfolio written on paper concerning practical activities.<br>Teacher's written assessment of student performance in practical activities (4 x per year).
Supervision level at the training stage	The student should be observed and accompanied over the two years and at the end of the second year:<br>The student is able to perform the EPA under reactive supervision, i.e., to perform the EPA autonomously and in an effective and safe manner with supervision readily available on request (supervision level 3).

Title	3.3 Follow-up of the Health Project in the Territory (HPT)
Short description	The student, in pairs of two, follows the implemented HPT, verifying community adherence to the community health needs care plan and analysing the results for continuous improvement and readjustment. Epidemiological data must be collected, and the HSP programme must be continued. A general overview of the territory must be maintained, and the obtained information must be entered into the DATA-SUS to store it securely.
Specifications	**To perform this EPA, the student:**<br>• Recognizes the care organization (work process/setting/policy).<br>• Communicates empathetically, respectfully, and ethically and is committed to the family's quality of life.<br>• Knows the territory, i.e., the geographic area of action of the FHS team, and recognizes its physical space, environmental protection and risk factors, sociodemographic profile, the educational e-health characteristics of the community and social resources.<br>• Develops clinical-epidemiological reasoning and evidence-based care plan evaluation.<br>• Defines the expected result and ways of measuring it during follow-up sessions.<br>• Monitors the implemented HSP and HPT, including their results, adherence, limitations, and potentialities.<br>• Follows territorial indicators. Considers epidemiological and clinical findings related to the adjustment of the community health plan and follows up on these data.<br>• Involves family members/the community in the plan discussion and evaluation.<br>• Discusses the HSP and HPT results as a team, namely, a multiprofessional health care team with intersectoral collaborators.<br>• Maintains updated information on the e-SUS AB/DATA-SUS and in patient and family medical records.<br>• Discusses his or her ideas with the student partner, the entire student group, the health team and the supervisor and identifies limitations and develops solutions.
Limitations	**This EPA does not apply to / a closer level of supervision is needed:**<br>**a.** The target beneficiaries have a risk of violence.
Knowledge, skills and attitude	**Knowledge:**<br>Recognize the care organization (work process/setting), health policy and care hierarchy; processes for referral and counterreferral; the principles of health surveillance, clinical epidemiology and epidemiological surveillance; concepts regarding life quality and health needs; evidence-based health care; the principles and techniques of communication; data documentation and index calculation; and data registration and interpretation. Comprehend the importance of the health care plan implementation and evaluation to community health quality. Know how to conduct a clinical-epidemiological reasoning and follow data of sentinel events. Know about referral and counterreferral procedures and mortality, morbidity, etc. Recognize the concepts of group collaboration and the promotion of multiprofessional and intersectoral professional integration. Understand the risks that are present in the care setting and territory. Know how to implement disaster management. Know local risks related to natural disasters.<br>**Skills:**<br>Form an overall impression of the community/community members and compare it with the data; engage in community-centred discussion; form a trusting professional-community/member and professional team relationship; recognize collective patterns and relevant abnormal findings; present a synthesis of the main findings and evidence to the supervising physician and the health team; identify evidence and engage in teamwork; register data in the community member or family medical record and on the e-SUS AB/DATA- SUS; and search the literature for qualified information related to the area health profile or to sentinel cases.<br>**Attitude:**<br>Open and respectful communication; attention to diversity (gender, age, and culture); use of language that is understandable to the team; ethical, respectful approach and management of the data and respect for the privacy and confidentiality of patient information; responsibility in health practice; commitment to the community/community member in question; reflection on the patient's and community's feedback on activities and team performance (attitudes and feelings) and health needs care plan evaluation and adjustment; recognition of the importance of the teams and that of sharing information with them; involvement and communication with patients and the community; and development of an embracement approach.
Conditions/implications of the entrustment decision	The continuous evaluation of the territory data and the implemented HPT and HSP can promote a functional community health care plan. The student must follow up on and analyse the quality of the offered health care, its comprehensiveness, community adherence, health education, and the potential for intervention. The follow-up presupposes commitment, teamwork, willingness on the part of the participants, adequate data records, evaluation of the plan and adjustment for adequacy. The supervisor must encourage the student, taking into account health surveillance data, to reflect on the community reality, to be creative and active and to compromise with respect to plan evaluation. The student must be encouraged to express his or her ideas and reference appropriate literature.
Domain of competence	Health surveillance/attention to health needs: integrality of care ranging from individual to collective needs, including the organization and management of care integrality.
Assessment sources	Reflexive portfolio written on paper concerning practical activities<br>Teacher's written assessment of student performance in practical activities (4 x per year).
Supervision level at the training stage	The student should be observed and accompanied over the two years and at the end of the second year:<br>The student is able to perform the EPA under reactive supervision, i.e., to perform the EPA autonomously and in an effective and safe manner with supervision readily available on request (supervision level 3).
